# Cross-Validation of Paranoid-Depressive Scale and Functional MRI: New Paradigm for Neuroscience Informed Clinical Psychopathology

**DOI:** 10.3389/fpsyt.2019.00711

**Published:** 2019-09-27

**Authors:** Drozdstoy Stoyanov, Sevdalina Kandilarova, Zlatoslav Arabadzhiev, Rossitsa Paunova, André Schmidt, Stefan Borgwardt

**Affiliations:** ^1^Neuropsychiatry and Brain Imaging Group, Department of Psychiatry and Medical Psychology, Plovdiv Medical University, Plovdiv, Bulgaria; ^2^Neuropsychiatry and Brain Imaging Group, Department of Psychiatry, University of Basel, Basel, Switzerland

**Keywords:** functional MRI, depression, schizophrenia, validation, psychopathology

## Abstract

There is reported a study performed with a novel paradigm aiming at investigation of the translational validity of von Zerssen’s paranoid-depression scale and its fMRI correlates in terms of focus on exploration of the results on the contrast between the Paranoid Specific (DP) blocks and the Depression Specific (DS) blocks. Patients with schizophrenia demonstrated significant activations in a number of regions (right angular gyrus, left posterior cingulate and precuneus, right transverse temporal gyrus) during responses to paranoia versus depression items which differ topologically from those found in patients with major depression (left middle cingulate and right superior temporal gyrus). The direct comparison between the groups, however, did not yield any residual activations after correction.

## Introduction

Considering the status of psychiatry as a hybrid discipline which embraces both the natural sciences and the humanities (1, 2), we attempt to deliver a novel, experimentally fostered concept of *translational validity*, which is a non-conventional and instrumentalist approach to validation (3).

As discussed in earlier publications (4–6) clinical and neurobiological measures are considered valid for different reasons *inside* their own divergent domains. All disciplines concerned with mental health establish internal or *intra-correlative validity*, i.e. psychological scales are typically validated against other psychological measures, and neurobiological measures are validated with other neurobiological tests. Psychiatric assessment tools represent a circle of validation between first-person measures (self-evaluation inventories) and third-person perspective, the psychiatric interviews (7). What is still missing is the *inter-correlative* or *inter-disciplinary validity which entails consistent inter-domain translation*. In practical terms the lack of consistent inter-domain translation is undermining the validity of psychiatric classifications as well as the implementation of the research findings in clinical practice (8).

Furthermore, we consider of critical importance the notion of “state dependence” as contrasted with the traditional “state independence” of biomarkers (9). State dependence means in this context that certain correlations are directly relevant and may be specific to the current mental state, yet not necessarily to the diagnosis in the medical sense. This is why the clinical and biological measures are beeing performed simultaneously in our paradigm (10).

The translation takes place on two levels according to the established psychometric vallidation standards: convergent and divergent (11). In first place the corresponding empirical measures are cross-validated on convergent level, e.g. depression clinical rating scale and blood oxygenation level dependent (BOLD) activation levels from functional magnetic resonance imaging (fMRI) during processing of neutral items in patient versus healthy control population (12). After that the construct is cross-validated in the same manner against another, presumably divergent clinical construct, e.g. paranoia. However discriminative power is not expected to be strong enough to underpin robust discrimination across nosological entities, but rather patterns of activation that may underpin the different psychopathological constructs and relevant measures.

In our previous studies we have tested the convergent validity by employing an fMRI paradigm using two types of visual stimuli—diagnostically specific (DS)—representing items from von Zerssen’s depression subscale (13) and diagnostically neutral (DN)—from an interest scale. Thus, we have been able to demonstrated that in healthy controls, contrasting the two types of stimuli (DS vs DN) yielded no significant brain activations, and the correlation analyses did not find a relationship between brain activations and the total score of the DS statements. On the other hand, in depressed patients contrasting the DS to the DN stimuli produced significant activations in several brain regions, and there were positive correlations with the DS score in several activation clusters (12, 14). In this manner, we were able to confirm the sensitivity of the method (its ability to distinguish healthy controls from depressed patients), still we had to address its specificity (different patterns of brain activation behind different clinical constructs and respective measures). As it has already been stated, such patterns are not likely to trascend to the level of nosological specificity.

To handle this last issue and to test the divergent validity, we further developed our paradigm with the specific aim of investigating the translational validity of von Zerssen’s paranoia-depression scale (13) and its fMRI correlates during their simultaneous implementation in patients with depression and schizophrenia.

## Methods

### Subjects

We recruited 35 psychiatric patients with either a diagnosis of schizophrenia (*n* = 15, mean age 37.1 ± 12 years, 11 males), or depressive episode (*n* = 20, mean age 42.3 ± 12.1 years, 5 males) in the context of major depressive disorder (*n* = 10, mean age 39.9 ± 11.9 years, 4 males) or bipolar disorder (*n* = 10, mean age 44.8 ± 12.5 years, 2 males). Subjects were assessed by an experienced psychiatrist (ZA) using the general clinical interview and the structured Mini International Neuropsychiatric Interview (M.I.N.I 6.0) (15) as well as the Montgomery–Åsberg Depression Rating Scale (MADRS) (16) and the Positive and Negative Syndrome Scale (PANSS) (17).

Patients were excluded if they had a comorbid psychiatric disorder (such as anxiety, substance related disorder), major medical illness, neurological disease, history of head trauma with loss of consciousness, or metal implants not compatible with the MRI. All participants provided a written informed consent complying with the Declaration of Helsinki and the study was approved by the University’s Ethics Committee.

### MR Scanning

The scanning of the participants was performed on a 3T MRI system (GE Discovery 750w). The MR protocol included high resolution structural scan (Sag 3D T1 FSPGR sequence), with slice thickness 1 mm, matrix 256 × 256, TR (relaxation time) 7.2 ms, TE (echo time) 2.3, and flip angle 12°, and a functional scan (2D EPI sequence), with slice thickness 3 mm, matrix 64 × 64, TR 2000 msec, TE 30 msec, and flip angle 90°. Before each functional scan 5 dummy time series were acquired.

### fMRI Stimuli and Procedure

We used a standard block-design with three different active conditions and one rest condition, with a total duration of 11 min and 44 s. Each active block lasted for 32 s and consisted of four text statements presented for 8 s each using NordicNeuroLab VisualSystem. For the Depression Specific (DS) blocks the statements were taken from the von Zerssen depression subscale (“I cry easily,” “I am more sensitive to criticism than I was before”), while for the Paranoia Specific (DP) blocks they were taken from the paranoid subscale (“Other people constantly follow and control me”). As in our previous study (12), there were also Diagnostically Neutral (DN) blocks consisting of four statements from a questionnaire about general interests and likes (such as “I like to write books or plays,” “I like to repair household appliances,” etc.). Under each written statement four possible answers (“completely true,” “mostly true,” “somewhat true,” “not true”) and the respective four response buttons (upper left, lower left, lower right, upper right) were presented. There were four blocks of each type, alternating between the three active conditions (DS, DN, and DP) followed by a 20 s resting block with a fixation cross in the middle of the screen (DS__DN__DP__D). For the active conditions, the participants were instructed to read the statements carefully and to respond with a button press according to their level of agreement, and for the passive condition, to focus on the fixation cross without thinking of anything in particular.

### fMRI Data Analysis

Data were analyzed using the SPM 12 (Statistical Paramertic Mapping, http://www.fil.ion.ucl.ac.uk/spm/) software running on MATLAB R2015 for Windows. The preprocessing included the following steps: i) realignment of the functional data for correction of head motion, ii) co-registration between the high-resolution anatomical image and the functional scans, iii) intra-individual estimation of spatial registration parameters based on the anatomical image, and iv) transformation of the co-registered functional data to standardized MNI (Montreal Neurological Institute) space, followed by v) spatial smoothing with a 8 mm full-width-at-half-maximum Gaussian kernel.

First-level analysis was conducted using a general linear model (GLM) applied to the time series, convolved with a canonical hemodynamic response function. Nuisance covariates included the six rigid body motion parameters. T-contrasts were defined for the active vs passive conditions. The resulting individual contrast maps from each comparison were then used in a second-level random-effects analysis to test for differences between the two patient groups (schizophrenia > depression and depression > schizophrenia). Furthermore, ANOVA design was used to explore the three clinical diagnosis—schizophrenia, major depression, and bipolar depression. The level of significance was set to p > 0.05 FWE (Family Wise Error) corrected using an uncorrected cluster-forming threshold of p < 0.001, and gender was used as a covariate in all second-level analysis.

Following the logic of our study in terms of differentiating between the clinical diagnosis of schizophrenia and depression by means of simultaneous application of fMRI and a clinical assessment tool (e.g. von Zerssen paranoia-depression scale), we focused our exploration of the results on the contrast between the Paranoia Specific (DP) blocks and the Depression Specific (DS) blocks.

## Results

### Demographic and Clinical Characteristics

The two patient groups did not differ significantly in their demographic and clinical characteristics (such as age, education, illness duration) except for the sex distribution, which was complying with the clinical reality, e.g. male prevalence in the schizophrenia group and the opposite for the depression group (**Table 1**).

**Table 1:** Demographic and clinical characteristics of all participants.

	Schizophrenia patients (*n*=15)	Depressed patients (*n* = 20)	Statistical significance
**Age** (mean ± SD)	37.1 ± 12	42.3 ± 12.1	0.210^a^
**Sex** (M/F)	11/4	5/15	*0.005^b^
**Education** (secondary/higher)	10/5	11/9	0.486^b^
**Age at onset** (years)	27.7 ± 8.2	32.1 ± 10.9	0.267^a^
**Illness duration** (months)	110 ± 95	140 ± 93	0.406^a^
**Episode duration** (weeks)	9.1 ± 7.1	15.3 ± 10	0.307^a^

SD, Standard Deviation. ^a^Independent samples t-test, ^b^ *χ* ^2^-test, *p < 0.05.

The two depression subsamples—unipolar and bipolar—were not significantly different in their demographics as well as in their clinical features.

### Comparative Analysis Across Schizophrenia and Depression Patients

**Figure 1:**
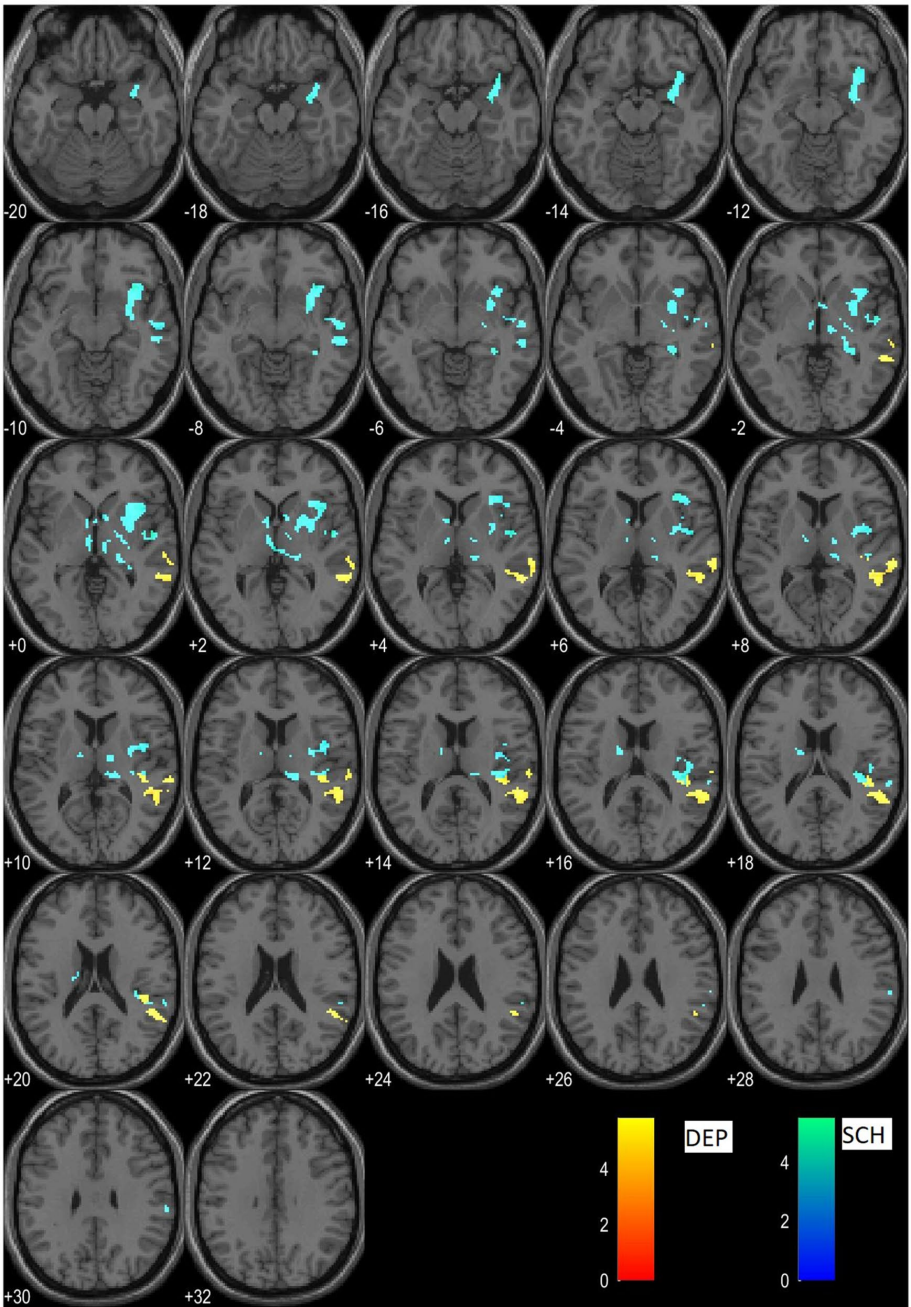
Clusters of residual activations of the DP > DS contrast in schizophrenia (cyan) and in depression (yellow).

The direct comparison of the DP > DS contrast between the two clinical populations produced multiple clusters of activation with significance level < 0.001 which did not survive above the 0.05 p-level after FWE correction. On the same inter-group level and prior to inclusion of gender as co-variate there was localized a cluster with sigin the right angular gyrus, with peak activation significance level p = 0.036, consistent with our findings on group level as described bellow. This cluster was above the level of significance after inclusion of gender as co-variate, which demonstrates the critical role of gender confound in such study design (discussed as limitation). On the group level (one sample *t*-test) the schizophrenic patients demonstrated residual activations in several clusters encompassing medial parietal and limbic structures (posterior cingulum and precuneus), as well as temporal and subcortical regions (for details see **Table 2**). The depressed patients, on the other hand, showed only two clusters with peak activations in middle cingulate and in superior temporal gyrus (**Table 2**). An illustration of these results is given in **Figure 1**.

**Table 2:** Clusters of significantly greater activations in schizophrenic and depressed patients when answering to psychosis items compared to depression items (DP > DS contrast).

Anatomical localization	Cluster size (voxels)	Peak MNI coordinates			p-value (FWE)
		x	y	z	
***Schizophrenic patients***					
**Right angular gyrus, SMG**	128	28	-46	36	0.004
**Left posterior cingulus** **and precuneus**	575	-6	-30	28	0.02
**Right transverse temporal gyrus and anterior insula**	3756	28	8	-10	0.05
**Right caudate, thalamus**	76	20	-12	22	0.05
***Depressed patients***					
**Left middle cingulate gyrus**	212	-8	-16	48	0.007
**Right superior temporal gyrus**	1243	42	-42	20	0.02

### Comparative Analysis Across Schizophrenia, Major Depression, and Bipolar Disorder

In addition, a one-way ANOVA model of the DP > DS contrast differentiated between the two depressed patients groups (unipolar and bipolar) and the schizophrenic group. Significant difference was found only between schizophrenic subjects and those with major depression in a single cluster located to the right pre/postcentral gyrus (201 voxels, p = 0.05 FWE) that is more activated in schizophrenia. The other between-group comparisons did not reach statistical significance. On the intragroup level, the schizophrenic subjects demonstrated residual activations almost identical to the ones revealed by the two-sample *t*-test, while the bipolar patients had only one cluster of greater activation located to the left middle cingulate gyrus (201 voxels, p = 0.012 FWE). No residual activations were found in major depression.

## Discussion

The main finding of our study maight be summarized as follows: patients with schizophrenia demonstrated significant activations in a number of regions (right angular gyrus, left posterior cingulate and precuneus, right transverse temporal gyrus) during responses to paranoia versus depression items (DP > DS contrast) which differ topologically from those found in patients with major depression (left middle cingulate and right superior temporal gyrus). The direct comparison between the groups, however, did not yield any residual activations after correction. The significance of these findings will be discussed in the following lines.

One of the clusters of activations, produced by the DP > DS contrast in schizophrenia, was in the area of the right angular gyrus, which belongs to the inferior parietal lobule system. It has been reported to be involved in semantic processing, social cognition, and reasoning as a cross-modal hub to converge multisensory information (18). Reversed assymetry in this region has been associated with schzophrenia (19) where abberant modulation/activation of the right angular gyrus was found as well (20).

Another cluster of residual activations in schizophrenic patients was stretching across the left posterior cingulate and precuneus which is implicated in autobiographical memory processing (21). It is assumed to contribute to episodic memory dysfunctions and abnormal functional connectivity that was found in schizophrenia (22, 23).

Amongst the significant clusters in our study, one was located in the right transverse temporal gyrus, or Heschl’s convolution and anterior insula. The function of those regions is related to accoustic processing as an *Inner voice*, or internal subjective dialogue with oneself (24), as well as task-level control of focal attention (25), which are often disturbed in schizophrenia.

On the other hand, the significant activations in the group of depressed patients were located in left middle cingulate gyrus as well as right superior temporal gyrus which makes sense in the context of the functional role of those regions in depression (26, 27).

Our ANOVA findings are consistent with previously reported results (12) and may be explained with the activation of the motor cortex as patients with schzophrenia used more often their left hand to provide positive responses to paranoid items.

The limited inter-group contrast in our findings (not reaching statistical significance) might be explained by the discrepancy of psychometric or psychodiagnostic versus diagnostic i.e. nosological validity. Psychometric validity essentally covers validation of particular construct(s) by use of another method (here, translational validation of von Zerssen paranoia-depression scale with fMRI), and it appears to have been achieved in our model. However the nosological validity assumes the possibility to validate entire medical–psychiatric–diagnostic entity and it remains out of reach.

The items that compose diagnostic scales, however precise those may be in order to measure certain phenomenon, could create preconditions for terminological inaccuracy. The diagnostic validity of a psychological tool can trace out borders of a particular category, but this is not enough to make a diagnosis. Even formally precise psychometric tools as intelligence and cognitive assessment tests can be challenged when their results are viewed in a specific emotional and cultural context (28, 29).

Another possible explanation of the overlap between the activations related to the processing of paranoid and depressive items in both patient groups might lay in the clinical variations of depressive symptoms in affective disorders and schizophrenia. The background of rather “warm” affectivity, induced by melancholia and anxiety in the context of affective disorder and the “cold” affect in schizophrenic psychosis (30, 31), caused by blunted affect might be revealed on phenomenological level (during the clinical interview) but cannot be captured properly by brief clinical assessment tools widely employed in psychopathology.

In addition, negative or cognitive symptoms in schizophrenic patients may be mistakenly assessed by a psychological tool as depressive (pseudodepresive) and vice versa. Morover, about 25% to 50% of the patients with major depression have impairment of at least one cognitive sphere (32). The most common disturbances in cognitive functioning during a depressive episode are those of memory, attention, and the degree of processing of various incentive stimuli (33). This is to demonstrate that cognitive deficits can be seen as a central element in the course of a major depressive disorder, not just as secondary phenomena (31). In the same perspective, similar changes in cognitive functioning can be found in schizophrenic patients in the context of negative and cognitive symptoms, and it would be impossible for a psychological test to differentiate them on the level of nosological specificity.

To summarize it there are limitations concerning possible *nosological specificity* of evaluation measures in clinical psychiatry, as predicted in some earlier theoretical publications (5, 8).

### Limitations

There are two major limitations which undermine generalizations from the current study.

The first is concerned with the small sample size, especially when the sample is subdivided into clinical diagnostic groups.

The second is the innovative and non-conventional approach to the the experimental paradigm design, which presents an issue for comparison with other studies in the field.

The third limitation apparently concerns the gender structure of the sample. In future replication studies gender balance will be of critical importance in order to bolster the significance of the results.

From a more general perspective those limitations might be addressed by expanding the research in translational neuroimaging using similar approach aiming to identify the functional MRI substrate behind clinical self-evaluation measures.

## Data Availability Statement

The datasets generated for this study are available on request to the corresponding author.

## Ethics Statement

The studies involving human participants were reviewed and approved by Research Ethics Committee, Plovdiv Medical University. The patients/participants provided their written informed consent to participate in this study.

## Author Contributions

DS has developed the conceptual rationale and delivered the text in the *Introduction* and a major part of the *Discussion*. SK has performed the statistical analysis and delivered the *Methods* and *Results* sections. ZA contributed to the interpretation of the *Results* and the *Discussion* sections. AS consulted the data analysis procedures and edited the manuscript. SB consulted the development of the paradigm and the data processing, and edited the paper and supervised the entire project. RP was involved in the empirical study and data processing.

## Conflict of Interest

The authors declare that the research was conducted in the absence of any commercial or financial relationships that could be construed as a potential conflict of interest.
